# The inhibiting role of hydroxypropylmethylcellulose acetate succinate on piperine crystallization to enhance its dissolution from its amorphous solid dispersion and permeability

**DOI:** 10.1039/c9ra08283b

**Published:** 2019-12-03

**Authors:** Yueyi Deng, Qi Liang, Yiru Wang, Xiaolan Zhang, Chengyun Yan, Yulin He

**Affiliations:** School of Pharmacy, Guilin Medical University 541004 Guilin Guangxi People's Republic of China dengyyqust@hotmail.com; School of Basic Medical, Guilin Medical University 541004 Guilin Guangxi People's Republic of China 418497164@qq.com

## Abstract

The purpose of this study was to demonstrate that inhibiting crystallization by HPMCAS played a key role in enhancing dissolution and absorption of piperine (Pip) from its amorphous solid dispersion (ASD). Nucleation induction time and supersaturation tests were used to evaluate the ability of the polymers to inhibit crystallization of Pip. The prepared solid dispersions were characterized by DSC and FTIR. The dissolution rate of Pip from its ASDs was assayed by a dissolution test. Pip permeability was investigated by single-pass intestinal perfusion studies. The order of the ability of polymers to inhibit Pip crystallization was HF > MF > LF > L100-55. The best inhibition effect of HF can be attributed to its hydrophobicity and steric hindrance. Pip is amorphous in polymer matrices when the ratio of Pip/HPMCAS is lower than 1 : 1 and Pip/L100-55 is lower than 3 : 1. IR spectra show that there are hydrogen bonds between the amide groups of Pip and the carboxyl groups of polymer. The order of the ability of polymers to enhance Pip dissolution is HF > MF > LF > L100-55, which coincided with the ability of polymers to inhibit Pip crystallization. Increased apparent permeability *via* HF-induced supersaturation and decreased apparent permeability *via* solubilization with L100-55 are demonstrated. Nucleation induction time and supersaturation tests may be used to screen appropriate polymers for preparing ASDs.

## Abstract 2

HPMCAS enhances piperine dissolution and permeability in amorphous solid dispersion by inhibiting crystallization.

## Graphical abstract

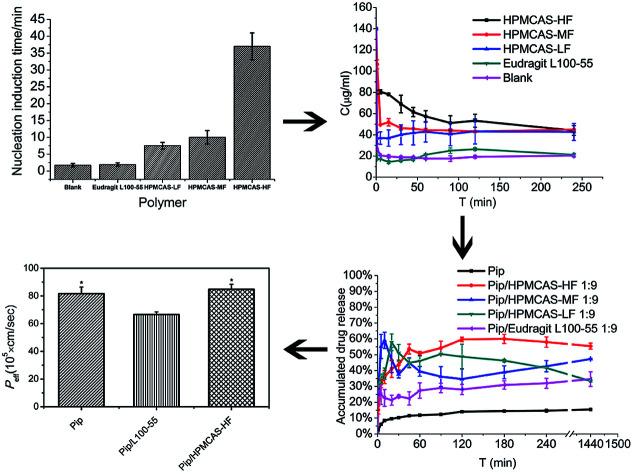

## Introduction

The number of poorly water-soluble candidate drugs has increased rapidly in the past few decades. It is estimated that more than 90% of the compounds in drug discovery pipelines are poorly water-soluble.^1,2^ Low solubility or dissolution rate will significantly reduce the absorption and oral bioavailability of a drug. Therefore, improving the solubility of the poorly water-soluble drugs has become one of the major challenges in the drug formulation field.^3,4^

Many formulation techniques, such as solid phase lipid nanoparticles, self-emulsifying delivery systems, formation of co-crystal and micelles, are being used to increase the solubility of the poorly water-soluble drugs.^5–7^ However, low drug loading limits the use of these technologies. In recent years, amorphous solid dispersion (ASD), which can increase the solubility of a large number of the poorly water-soluble drugs with different physical and chemical properties, has aroused strong concern.^8–10^

ASD is defined as a situation where one or more than one drug is dispersed in polymer matrix and exhibits amorphous state in the solid dispersion. The amorphous drug with high energy has larger solubility compared to its crystal form. ASD has high drug loading, and the water-soluble polymer matrixes used for ASD are derived from pharmaceutical excipients with high safety. It has been reported that the solubility enhancement by solubilizer reduces the transmembrane transport of drug, but the supersaturated solution (the apparent solubility of drug, *C*_app_, is higher than the equilibrium solubility, *C*_S_) formed by dissolution of ASD, enhances drug transmembrane transport for its higher chemical potential.^11–13^ Unfortunately, the supersaturated solution tends to crystallize due to its high energy state. The extensive research have shown that some water-soluble polymer excipients, such as HPMCAS, HPMC and PVP, play a key role on maintaining the supersaturated solutions as precipitation inhibitors.^14–17^ Therefore, the rational selection of polymer used for ASD is very important for developing ASD formulation successfully.

Pip is an alkaloid and extracted from black pepper. It has exhibited anti-inflammatory, antioxidant, anti-tumor and antidepressant activities.^18–20^ In addition, it is an inhibitor of glucuronidation and an P-gp efflux transporter located on the gastrointestinal epithelial cells, which can be used to improve the bioavailability of many drugs. It has been reported that Pip can effectively improve the bioavailability of curcumin as an oral absorption enhancer recently.^21–23^ As a drug, Pip is not able to reach a high concentration in the gastrointestinal tract due to its low solubility and rapid crystallization in aqueous solution, so its therapeutic role is limited.^24^

In this study, the water-soluble polymers HPMCAS with different carboxyl content (HF, MF, and LF) and Eudragit L100-55 were chosen as matrixes to prepare the amorphous solid dispersions of Pip. Pip is weak base and the polymers with carboxyl groups show acidity in aqueous media, so there will be acid–base interactions between Pip and the polymers. These polymers may maintain supersaturated Pip solution through these interactions and enhance dissolution of Pip. By measuring the induction time of nucleation and supersaturation test, the effect of inhibiting Pip crystallization by these polymers was investigated. The ASDs of Pip were prepared by the solvent evaporation method and characterized by DSC and IR. The dissolution profiles of the ASDs of Pip were investigated by dissolution test. Permeability of Pip solution in the absence or presence of polymer was assayed by *in situ* single-pass intestinal perfusion studies.

## Materials and methods

### Materials

Pip (97%) was purchased from Sinopharm Chemical Reagent Co., Ltd (Shanghai, China). HF (degree of substitution (DS) (acetyl) = 0.67, DS (succinoyl) = 0.18), MF (DS (acetyl) = 0.52, DS (succinoyl) = 0.26) and LF (DS (acetyl) = 0.48, DS (succinoyl) = 0.37) were obtained from Shin-Etsu Chemical Company (Tokyo, Japan). L100-55 (1 : 1 methacrylic acid and methyl methacrylate copolymer) was donated by Evonik Industries AG (Darmstadt, Germany). All other reagents were analytical grade and used as received.

### Solubility measurement

The selected polymers were predissolved in 100 mM pH 6.8 PBS at a concentration of 0.63 mg mL^−1^. The equilibrium solubility of Pip was determined in the absence and presence of polymer. An excess amount of Pip was added to 20 mL PBS and stirred at 350 rpm and 37 °C for 24 h. After filtration through a 0.22 μm cellulose ester membrane filter, the drug concentration in the filtrate was obtained by HPLC.

### Supersaturation test

**Fig. 1:**
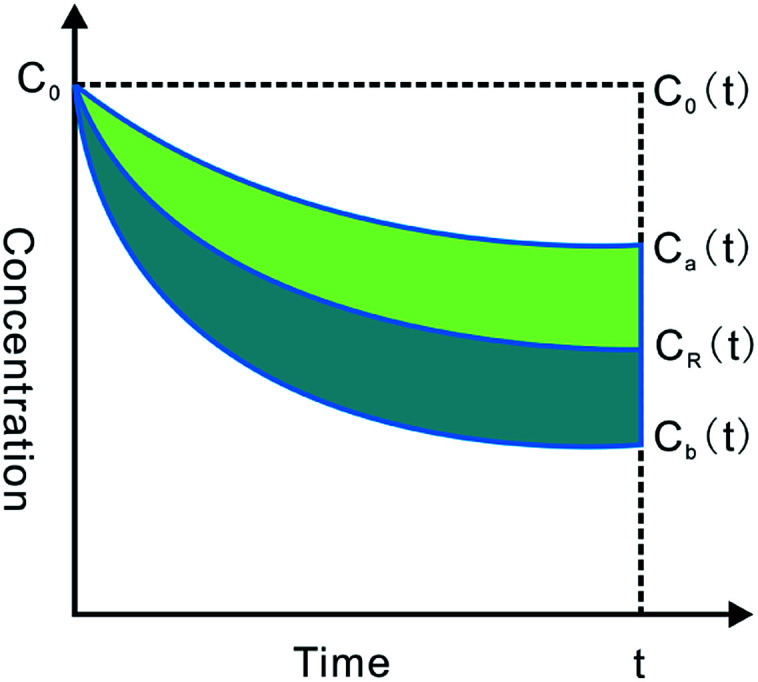
Schematic diagram of supersaturated parameters.

200 μL DMSO solution of Pip (56 mg mL^−1^) was added to 80 mL pH 6.8 PBS with or without polymer (0.63 mg mL^−1^) under stirring at 37 °C. The concentration of DMSO was very low and had no effect on the solubility of Pip. 3 mL of solution was sampled and filtered through 0.22 μm cellulose ester membrane at 5, 10, 15, 30, 45, 60, 90, 120 and 240 min after mixing. The concentration of Pip was determined by HPLC. The ability of polymer to maintain supersaturation could be evaluated by supersaturation parameters (SSP).^25^ As shown in Fig. 1, *C*_0_ is the initial concentration of Pip, and *C*_R_(*t*) is Pip concentration at the *t* moment in the absence of polymer. *C*_a_(*t*) or *C*_b_(*t*) is Pip concentration at the *t* moment in the presence of polymer. SSP can be calculated by the following formula:
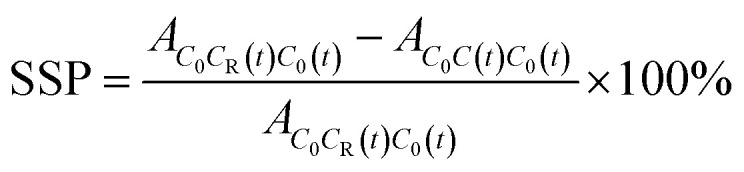


### Nucleation induction time measurement

Nucleation induction time (*t*_ind_), which is used to characterize crystallization kinetics, is defined as the required time that the small crystals with a detectable size begin to form in supersaturated solution. *t*_ind_ can be determined by the abrupt increase in intensity of light scattered (extinction) from drug solution. Extinction at 430 nm for Pip is detected by a UV/vis spectrometer (UV-1200, Shimadzu, Japan) and Pip has no absorbance at this wavelength. Supersaturated drug solutions were prepared by the solvent-shift method. 0.5 mL DMSO solution of Pip (4 mg mL^−1^) was added to 200 mL pH 6.8 PBS in the absence and presence of predissolved polymers (0.63 mg mL^−1^) under stirring at 350 rpm. After mixing, 0.5 mL solution was sampled and measured by the UV/vis spectrometer at a certain time interval.

### Preparation of solid dispersions

Pip/polymer mixtures (1 g, w/w: 3 : 1, 1 : 1, 1 : 3 and 1 : 9) were dissolved in 100 mL of ethanol/dichloromethane mixtures (v/v: 1 : 1). The solvent was removed using rotary evaporation at 60 °C and the remaining solid was dried in vacuum for 24 h. Solid dispersions were ground with a mortar and pestle and passed through a 60 mesh sieve.

### Differential scanning calorimetry (DSC)

The thermal analysis was carried out using a DSC (214, Netzsch, Germany). A 2–5 mg solid dispersion sample was loaded in an aluminum pan and sealed by a lid with a pin hole, taking an empty aluminum pan as reference. Samples were heated from 35 °C to 200 °C at a heating rate of 10 °C min^−1^ in an atmosphere of nitrogen with a flow rate of 20 mL min^−1^. DSC curves were analyzed using Proteus software (Netzsch, Germany).

### FTIR spectroscopy

FTIR spectra were recorded in the range of 400–4000 cm^−1^ on a Nicolet 170SX spectrometer with pressed KBr pellets. 128 scans were collected for each sample, and the resolution was 4 cm^−1^.

### Dissolution studies

Pip release profiles were investigated under non-sink conditions. 320 mg Pip/polymer 1 : 9 ASDs were added to 200 mL pH 6.8 PBS with stirring at 150 rpm and 37 °C. Samples of 2 mL were withdrawn at predetermined time intervals (1, 5, 10, 20, 30, 45, 60, 90, 120, 180, 240 min and 24 h) and replaced with 2 mL fresh dissolution medium. The aliquots were filtered through 0.22 μm cellulose ester membrane and Pip concentration in the filtrate was determined by HPLC. Experiments were performed in triplicate and the average values with standard deviations were presented for all dissolution profiles.

### *In situ* single-pass intestinal perfusion studies

All animal experiments were performed in compliance with the NIH guidelines for the Care and Use of Laboratory Animals (NIH Publication, Eighth edition, 2011) and were approved by the Experimental Animal Ethics Committee of the Guilin Medical University (Guangxi, China). Male albino Sprague-Dawley rats weighing 250–300 g were used for all perfusion studies. Prior to each experiment, the rats were fasted for 12–18 h with free access to water.

The experimental procedure for the single-pass perfusions followed previous reports.^26–28^ Rats were anesthetized by 1 mL kg^−1^ ketamine–xylazine solution (9% : 1%) and placed alongside of a infrared light to keep normal body temperature. A 3–4 cm incision was made along the midline of abdomen. A proximal jejunal segment of approximately 10 cm was carefully exposed and cannulated on two ends with flexible PVC tubing. The exposed intestinal segment was kept moist with the wetting wound dressing.

The supersaturated solution (3 times equilibrium solubility, according to the ability of HF to inhibit crystallization) were prepared by adding 4 mg mL^−1^ DMSO solution of Pip to 200 mL 50 mM pH 6.8 PBS containing HF (0.63 mg mL^−1^) under stirring at 350 rpm. Pip was dissolved in 200 mL 50 mM pH 6.8 PBS containing L100-55 (0.63 mg mL^−1^) to obtain the concentration of 60 μg mL^−1^. All perfusate solutions were kept at 37 °C in a water bath and were pumped through the isolated intestinal segment (HL-2, HUXI, China). The intestinal segment was first rinsed with blank perfusion buffer at a flow rate of 0.5 mL min^−1^ until any residual debris were cleaned out. The perfusate solutions containing Pip were then pumped through the intestinal segment for 1 h at a flow rate of 2.5 mL min^−1^ in order to reaching steady state conditions. In another hour, samples were collected at an interval of 15 min in a pre-weighed glass tube. Drug concentration in perfusion samples at different time points and original drug solution were immediately assayed by HPLC. At the end of the experiment, animals were euthanized with saturated potassium chloride solution by intracardiac injection. The length and radius of each intestinal segment was accurately measured.

Water absorption was determined by the gravimetric method. The outlet concentration of Pip was corrected by the following equation:
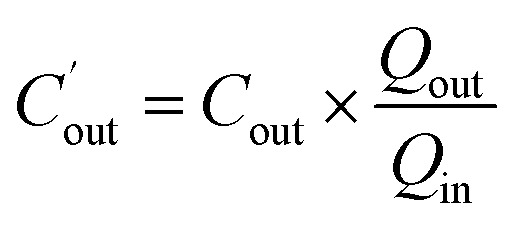
where 
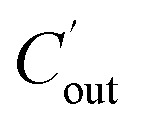
 is the corrected outlet concentration of Pip; *C*_out_ is the outlet concentration of Pip; *Q*_out_ and *Q*_in_ are the outlet and inlet perfusate flux, respectively.

The effective permeability (*P*_eff_) through the rat gut wall was determined by the following equation:
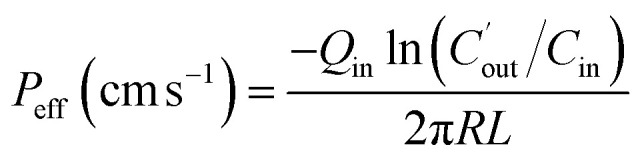
where *Q*_in_ is the inlet perfusate flux; *C*_in_ is the inlet concentration of Pip; *R* is the radius of the intestinal segment, and *L* is the length of the intestinal segment.

### High performance liquid chromatography (HPLC)

Pip concentration was determined using an Agilent 1100 HPLC system (Agilent Technologies, Santa Clara, CA). Pip was detected by UV absorbance at 343 nm. A Zobrax SB-C18 column (150 × 4.6 mm^2^, 5 μm) was applied to chromatographic separation. The mobile phase consists of phosphate buffer (pH 6.8) (30%) and methanol (70%). The flow rate was 1 mL min^−1^ and the injection volume was 20 μL.

### Statistical analysis

All *in vitro* experiments were performed in triplicate, and all animal experiments were *n* = 4. The data are presented as the mean ± the standard deviation (SD). Statistically significant differences of *P*_eff_ were determined by analysis of variance (ANOVA) and Tukey tests, and a *p* value of less than 0.05 was termed significant.

## Results and discussion

### Effect of polymer on equilibrium solubility of Pip

**Fig. 2:**
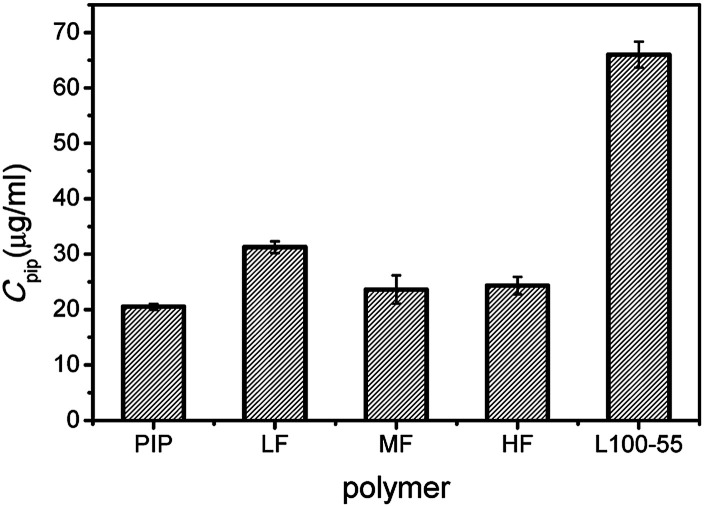
The equilibrium solubility of Pip in the presence or absence of polymers.

The equilibrium solubility of crystalline Pip is 20 μg mL^−1^, and the apparent solubility of Pip increases in the presence of polymer (Fig. 2). The selected polymers have solubilization effect on Pip, and the order of solubilization is L100-55 > LF > MF ≥ HF. There is a direct correlation between solubilization ability and carboxyl content in polymer (the order of carboxyl content of polymer: L100-55 > LF > MF > HF). These results indicate that there is an acid–base interaction between polymer and Pip, which improves the equilibrium solubility of Pip.

### The maintaining role of polymer on the supersaturated solution of Pip

**Fig. 3:**
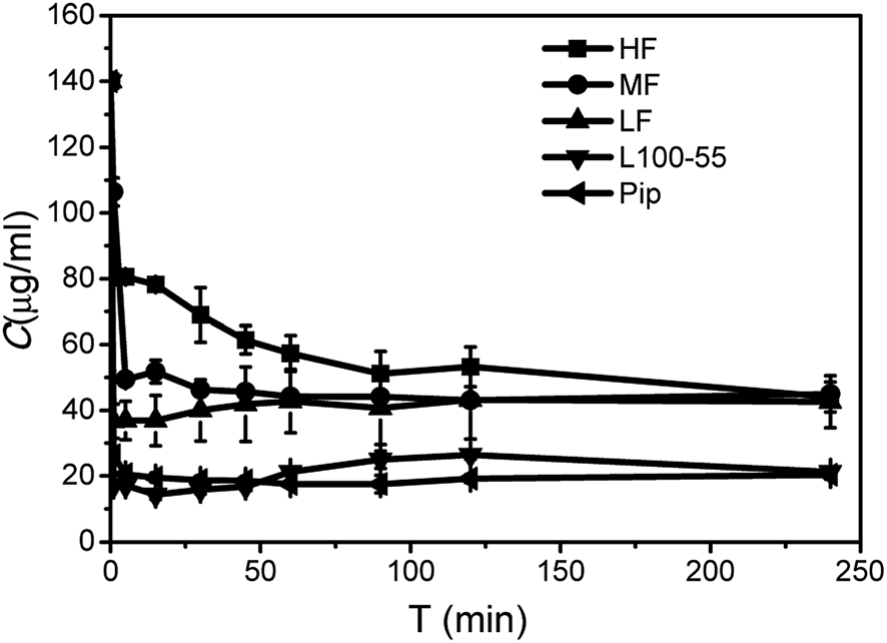
Desaturated curves of Pip supersaturated solutions in the presence or absence of polymers.

As shown in Fig. 3, Pip precipitated rapidly and the concentration decreased to 26.4 μg mL^−1^ in 1 min, indicating that almost no supersaturation was formed. SSP can be used to evaluate the ability of polymer to maintain supersaturation. From Table 1, the order of stabilizing Pip supersaturation solutions is HF > MF > LF > L100-55, which is opposite to the solubilization role. L100-55 can enhance Pip solubility, but it has little role on maintaining supersaturation. It can be seen from Fig. 3 that HPMCAS can inhibit crystallization of Pip from the supersaturated solutions and significantly prolong supersaturation time, acting as “parachutes”. The inhibition role of polymer is carried out by thermodynamics or kinetics.^29,30^ The thermodynamic way can reduce the crystallization rate by a decrease of supersaturation degree with improving solubility. Kinetics inhibition refers to the reduced rates of nucleation and crystal growth. According to solubility studies, HPMCAS has a little effect on Pip solubility, so the crystallization inhibition role of HPMCAS is based on kinetics. The factors affecting the inhibition ability of polymer include drug–polymer interaction, steric hindrance of polymer, hydrophobicity and solution viscosity, *etc.*^31–33^ HPMCAS has a better inhibition role than L100-55, which may be ascribed to steric hindrance of HPMCAS. HF is the most effective in the inhibition of crystallization of Pip, which may be attributed to the strong hydrophobic interactions between Pip and HF (HF is the most hydrophobic when compared to MF and HF due to its low carboxyl content).

**Table 1:** SSP values in the supersaturation test

SSP/%				
	MF	HF	LF	L100-55
Pip	22	30	19	1.4

**Fig. 4:**
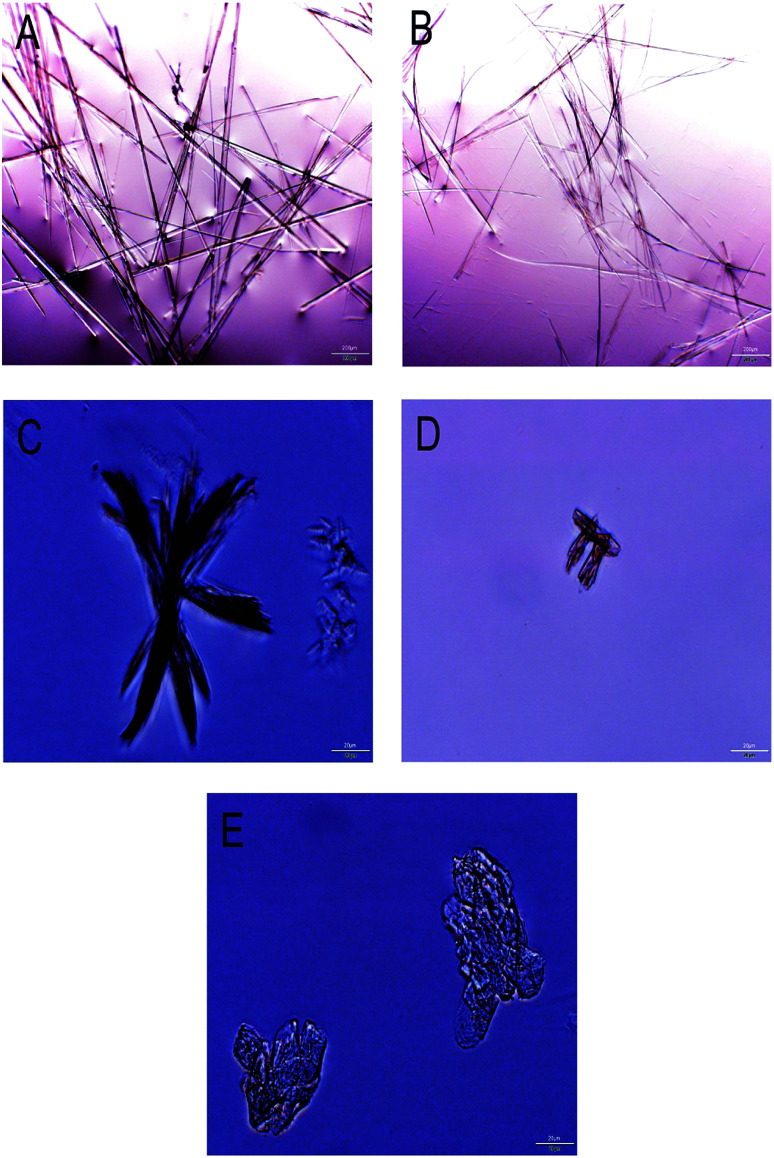
Optical microscopic images of Pip crystal in the presence and absence of polymers ((A) no polymer; (B) L100-55; (C) LF; (D) MF; (E) HF).

Following the supersaturation experiment, the precipitate was investigated by optical microscopy. Pip crystals have long needle to rod-like morphology with high aspect ratio in the absence of polymer (Fig. 4A). The crystal morphology doesn't change in the presence of L100-55 (Fig. 4B), indicating L100-55 has little effect on the process of nucleation and crystal growth. This result is in agreement with the supersaturation result. Pip crystal surfaces become rough and have lower aspect ratio in the presence of HPMCAS (Fig. 4C–E). Rough surfaces reveal that crystals grow by diffusion mechanism, in which the diffusion of Pip molecules is limited by large steric hindrance of HPMCAS. Pip crystals have plate-like morphology and the lowest aspect ratio in the presence of HF, indicating that there are the strongest interactions between Pip and HF.^34^ The extent to which the aspect ratio of crystals is modified correlates with the crystallization inhibition ability of these polymers.

### Inhibition of nucleation process of Pip by polymer

**Fig. 5:**
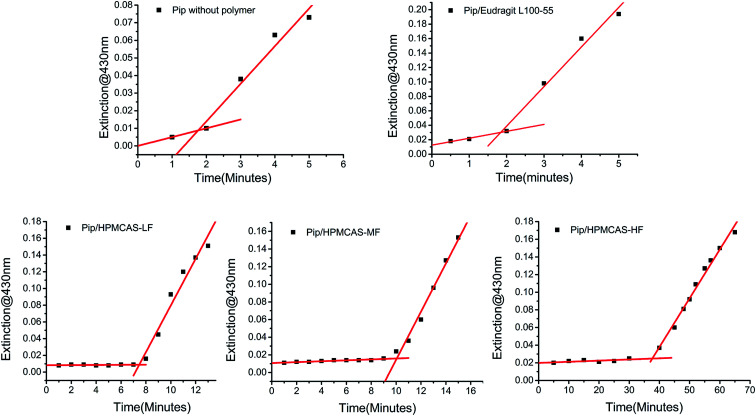
The change of extinction with time of Pip supersaturated solutions in the presence or absence of polymers.

Nucleation behavior of Pip from the supersaturated solution was investigated in the presence or absence of polymer. Nucleation can be quantified by measuring the time that small crystal particles begin to be detected, which is known as *t*_ind_. As shown in Fig. 5, the *t*_ind_ of Pip nucleation is 2 min in the absence of polymer and 5, 8, 10, 40 min in the presence of L100-55, LF, MF and HF, respectively. So the order of inhibiting nucleation ability of polymer is HF > MF > LF > L100-55. This order is consistent with the ability of polymer to maintain Pip supersaturated solution, which suggests that the inhibition of polymer on Pip crystallization may be ascribed to the inhibition of nucleation process of crystallization. The ability of nucleation inhibition of polymer is determined by drug–water–polymer interaction.^35^ It is very important that polymer has similar hydrophobicity to drug for nucleation inhibition. Too low or high hydrophobicity of polymer is unfavourable to nucleation inhibition.^36^ HF has the most effective inhibition on Pip nucleation, indicating that HF has close hydrophobicity to Pip. The stronger hydrophobic interaction between Pip and HF disrupt Pip aggregates, which can further assemble into small crystals with order structure. The nucleation inhibition ability of LF, MF and HF is higher than that of L100-55, which indicates that HPMCAS with large steric hindrance can prevent the assembly of Pip molecules more effectively than L100-55.

### Characterization of the ASDs

**Fig. 6:**
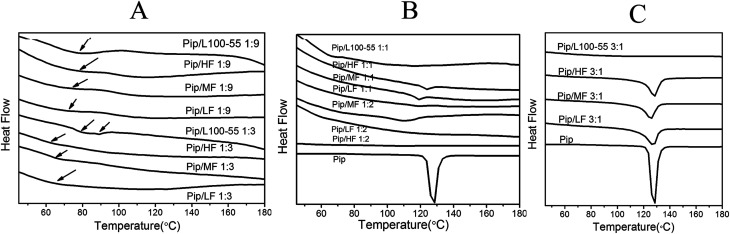
The DSC curves of 1 : 9 and 1 : 3 Pip solid dispersions (A); the DSC curves of 1 : 1 Pip solid dispersions (B); the DSC curves of 3 : 1 solid dispersions (C).

The DSC curves of the prepared solid dispersions are shown in Fig. 6. As shown in Fig. 6B, pure Pip has one melting peak at 131.1 °C. In Fig. 6A, there are no melting peaks of Pip for the 1 : 3 and 1 : 9 Pip/polymer solid dispersions, which indicates that Pip is dispersed amorphously in polymer and the ASDs of Pip are obtained. If a solid dispersion shows only one *T*_g_, then the drug and the polymer have good compatibility.^37–39^ All the 1 : 3 and 1 : 9 Pip/HPMCAS solid dispersions show one *T*_g_, while 1 : 3 Pip/L100-55 has two *T*_g_s, indicating that HPMCAS has better compatibility with Pip than L100-55. The two *T*_g_s of Pip/HF solid dispersions are closer to each other than those of the Pip/MF and Pip/LF solid dispersions, which suggest that stronger interactions exist between HF and Pip. Two *T*_g_s of 1 : 3 Pip/L100-55 solid dispersion are closest to each other, which may suggest that Pip is distributed better in L100-55 than HPMCAS during preparation for good solubility of L100-55 in the solvents.

The drug loading capacity of solid dispersion is the maximum amount of drug that can be amorphous in polymer carrier.^40^ The 1 : 2 solid dispersions of HF, MF and LF have no endothermic peaks and the 1 : 1 solid dispersions show melting peaks between 120 °C and 130 °C (Fig. 6B), which indicates that the maximum drug loading of HF, MF and LF is between 33% and 50%. The 3 : 1 solid dispersion of L100-55 has a very small melting peak at 126.2 °C (the peak can not be seen in Fig. 6C) and there is no endothermic peak when the ratio of Pip and L100-55 is 1 : 1 (Fig. 6B), which shows that the maximum drug loading of L100-55 is close to 75%. The melting peak area of 1 : 1 solid dispersions of HF, MF and LF are 1.56, 1.381 and 0.4073 J g^−1^ respectively, which shows that the order of drug loading abilities of polymers under the same condition of preparation is L100-55 > LF > HF > MF. L100-55 has the highest drug loading capacity may be attributed to better Pip distribution in L100-55 than HPMCAS during preparation for good solubility of L100-55 in the solvents. The melting points of solid dispersions decrease compared with pure Pip (Fig. 6B and C), indicating that there are interactions between Pip and polymers, which disrupt crystallization of Pip.^41^ With the increase in drug content, the melting points of solid dispersions are closer to that of Pip.

**Fig. 7:**
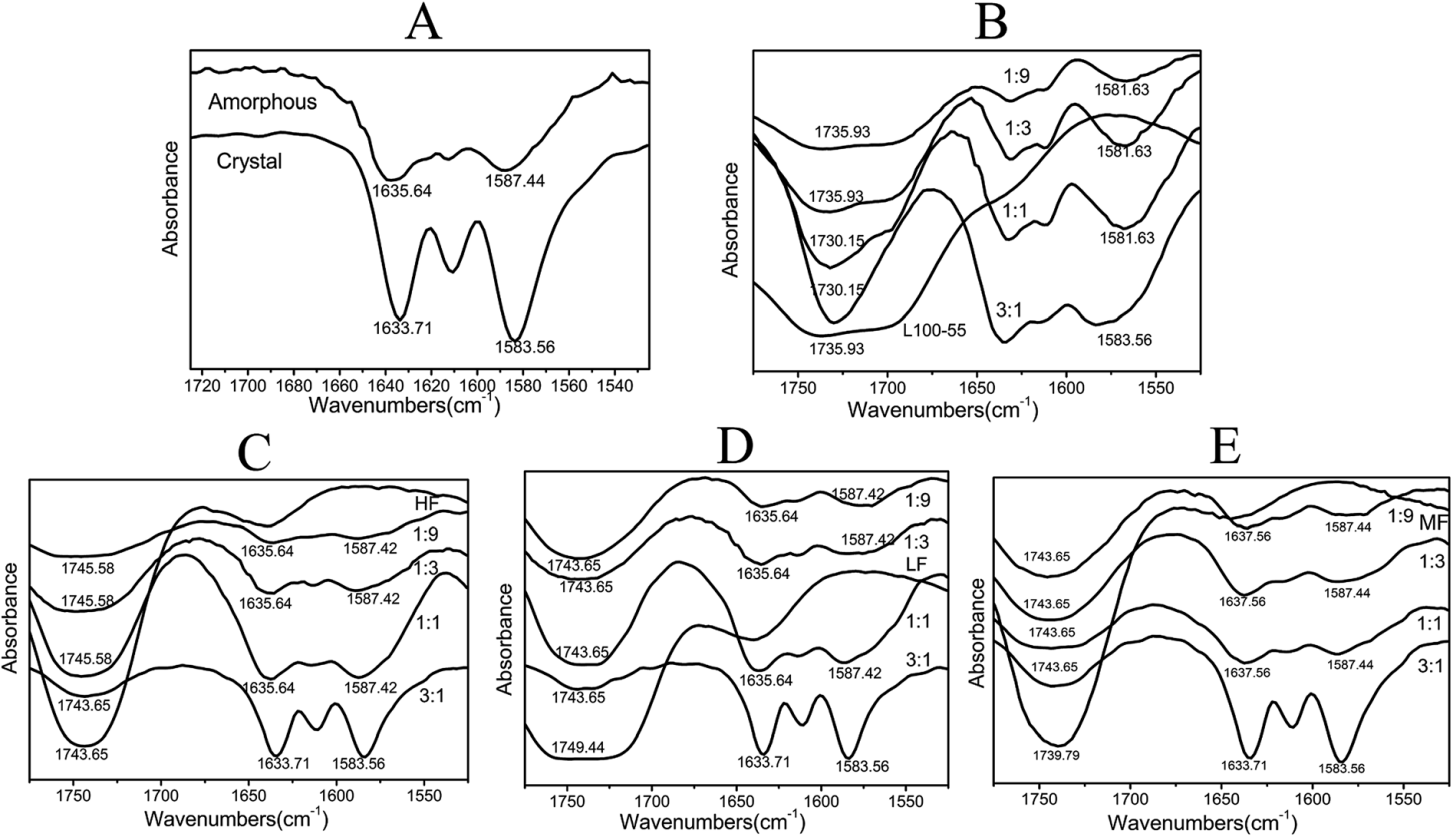
(A) is IR spectra of crystal Pip and amorphous Pip; (B), (C), (D) and (E) are IR spectra of Pip/L100-55, Pip/HF, Pip/LF, Pip/MF solid dispersions with different proportions respectively.

The FTIR spectra of dispersion solids are shown in Fig. 7. For crystalline Pip, the strong band at 1583 cm^−1^ is ascribed to benzene ring stretching and the peak at 1634 cm^−1^ is C=O–N absorption. For amorphous Pip, these peaks shift to 1587 and 1636 cm^−1^ respectively, which shows the change of the molecular environment of these groups. The bands of amorphous Pip are more smooth and broad than those of crystalline Pip. When the ratio of drug and polymer (HF, MF and LF) is smaller than 3 : 1, the shape and wavenumber of the peaks of the solid dispersions are similar to those of amorphous Pip, suggesting that these solid dispersions are amorphous. The 3 : 1 solid dispersions of HF, MF and LF exhibit the bands of crystalline Pip at 1583 and 1634 cm^−1^, showing the presence of crystalline Pip in these solid dispersions. These results are in agreement with the DSC results. The peaks around 1740 cm^−1^ are absorption bands of polymer carbonyl groups and these peaks shift compared to pure polymers, which confirms Pip–polymer interactions.^42^ For Pip/L100-55 solid dispersions, the shoulder peaks at 1715 cm^−1^ disappear and the peaks around 1700–1740 cm^−1^ become stronger as drug content increase, indicating stronger Pip–polymer interactions.

### Drug release profiles

**Fig. 8:**
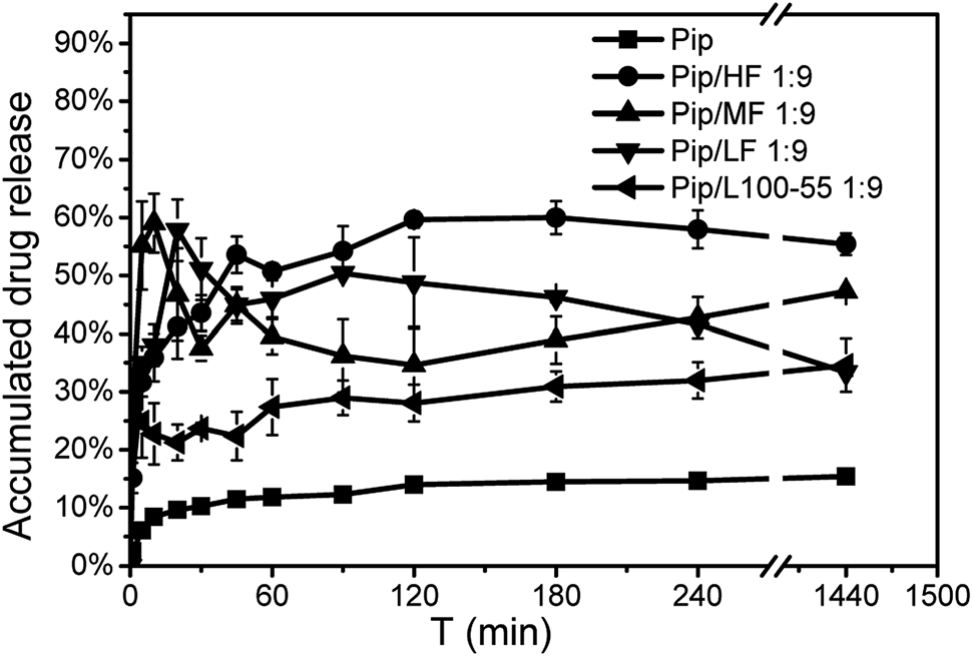
Dissolution curves of Pip, the 1 : 9 Pip/L100-55 and Pip/HPMCAS solid dispersions in pH 6.8 PBS.

As shown in Fig. 8, the prepared ASDs can enhance the dissolution of Pip. The dissolution rate of pure Pip was very slow and only 14.5% of Pip release was reached in 3 h. The dissolution rate and drug release of the ASDs were higher than that of pure Pip. During dissolution, we found that the ASDs were immediately mixed with the dissolution medium and the solution became cloudy, while the pure Pip floated on the surface of the dissolution medium for a long time. These results indicate that the polymer carriers enhance the wettability of Pip and improve Pip release.^43^ Release from the Pip/MF, Pip/LF and Pip/L100-55 ASDs was very fast, reaching 59% within 10 min, 57.8% within 15 min and 32% within 5 min respectively, while release from the Pip/HF ASD was slow and reached 59.6% within 3 h. These results may be related to the dissolution rate of polymer, the order of which in the dissolution medium is L100-55 > LF > MF > HF. It can be found from Fig. 8 that L100-55, LF and MF can accelerate the dissolution rate of Pip, but these polymers can not maintain a high Pip concentration in 24 h. HF can not only increase the dissolution rate of Pip, but also maintain a higher drug concentration within 24 hours. We can obtain the order of the ability of polymer to enhance the dissolution of Pip is HF > MF > LF > L100-55, which is consistent with the ability of polymer to inhibit Pip nucleation and maintain the supersaturated solution of Pip. These results indicate that inhibiting Pip crystallization by polymer plays a key role for enhancing Pip dissolution.

### Effect of supersaturation on permeability

**Fig. 9:**
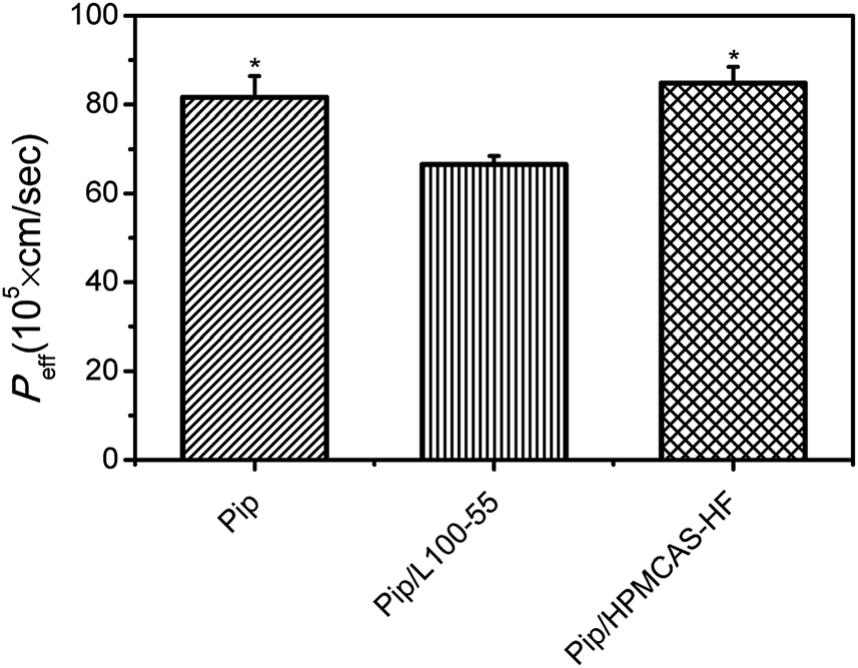
*P*_eff_ of from saturated solution, a solution containing L100-55 and a supersaturated solution in the presence of HF was assayed by single-pass intestinal perfusion studies. *p < 0.05 *versus* Pip solution. Average ± SD; *n* = 4.

Pip permeability from saturated solution (20 μg mL^−1^), a solution containing L100-55 (60 μg mL^−1^) and a supersaturated solution in the presence of HF (60 μg mL^−1^) was investigated by single-pass intestinal perfusion studies. As shown in Fig. 9, the apparent permeability of HF-induced supersaturated solution remained unchanged compared to Pip saturated solution, whereas Pip solution in the presence of L100-55 resulted in decreased apparent permeability. As a result, overall flux increased markedly with increasing apparent solubility *via* supersaturation. It had been reported that increasing apparent solubility *via* cyclodextrins, surfactants, and cosolvents resulted in decreased apparent permeability.^28,44,45^ These results show that the solubilization mechanism may affect intestinal absorption. Solubilizers such as cyclodextrins, surfactants, and cosolvents increase aqueous solubility of drug by complexation and micellization, which result in decreased “free” drug available for absorption. As for supersaturation, the high apparent solubility is maintained by kinetically inhibiting crystallization with polymer, resulting in increased “free” fraction of drug with high chemical potential to enhance transmembrane transport. Recently, some polymers with solubilization effect, such as PVP, have been found to decreases the apparent permeability.^46^ In our studies, we obtain the similar result that L100-55 with good solubilization role on Pip sacrifice apparent intestinal membrane permeability, which may be attributed to the encapsulation of the drug into L100-55 and the change of the molecular state of Pip in the presence of the acid–base interaction.^46^

## Conclusions

In this paper, HF, MF, LF and L100- 55 were used as carriers to prepare ASDs of Pip. Dissolution enhancement by the Pip/HF solid dispersion can be own to the strong nucleation inhibition ability of HF. Compared with the acid–base interaction, hydrophobicity and steric hindrance of polymer are the dominant factors for maintaining the supersaturated solution of Pip. HF-induced supersaturation resulted in increased apparent intestinal membrane permeability, whereas solubilization by L100-55 resulted in decreased apparent permeability. Nucleation induction time and supersaturation test may be used to screen appropriate polymers for ASD formulations.

## Conflicts of interest

There are no conflicts to declare.

## Supplementary Material
